# Supplementary material for: Hippocampal seed connectome-based modeling predicts the feeling of stress
Source: Nat Commun. 2020 May 27;11:2650. doi: 10.1038/s41467-020-16492-2 (PMC7253445; doi:10.1038/s41467-020-16492-2)
Supplement: Supplementary file 1 — Supplementary Information [file 41467_2020_16492_MOESM1_ESM.pdf]

Supplementary Information

Hippocampal seed connectome-based modeling predicts the feeling of stress

Goldfarb et al

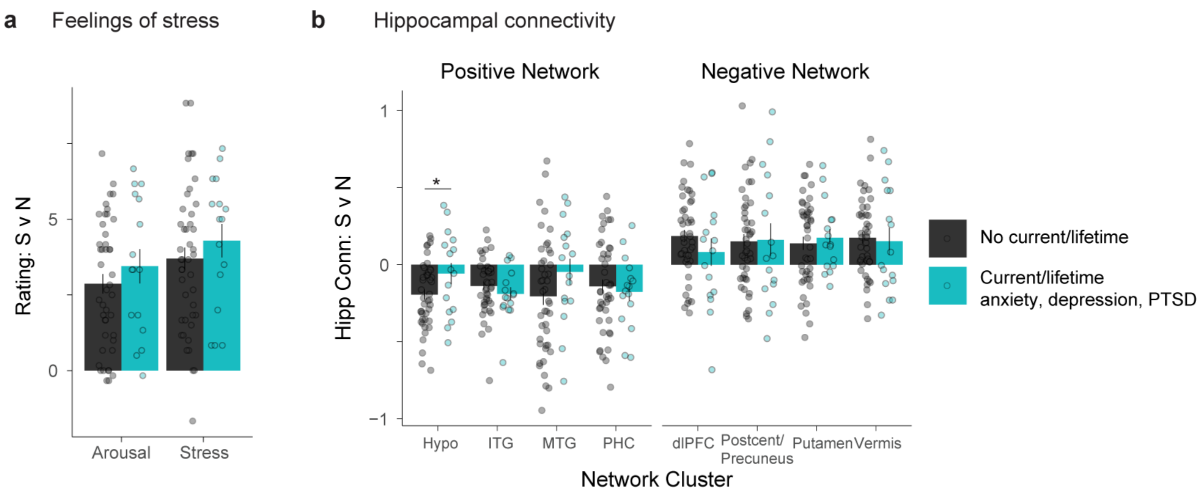

Supplementary Fig. 1. Stressor responses in participants with psychiatric history. **a,** Participants meeting either current or lifetime criteria for anxiety, depression, or posttraumatic stress disorder (PTSD; SCID-5 criteria; shown in turquoise, N = 16) did not self-report significantly higher ratings of stress or arousal during the stressor (S) relative to the neutral condition (N) compared to participants without these diagnoses (gray, N = 44). Ratings were compared using two-sided independent samples *t*-tests (both $p > .25$). **b,** Overall, stressor-modulated hippocampal connectivity with predictive clusters did not significantly differ with psychiatric diagnoses (all except hippocampal/hypothalamus $p > .14$). However, participants meeting criteria for these diagnoses did have significantly higher hippocampal/hypothalamus connectivity ($p = .049$), suggesting a less adaptive response during the stressor. Connectivity was compared between groups using two-sided independent samples *t*-tests. As these analyses are exploratory, they were not corrected for multiple comparisons. Source data are provided as a Source Data file. Error bars = $\pm$ 1 SE. *$p < .05$.

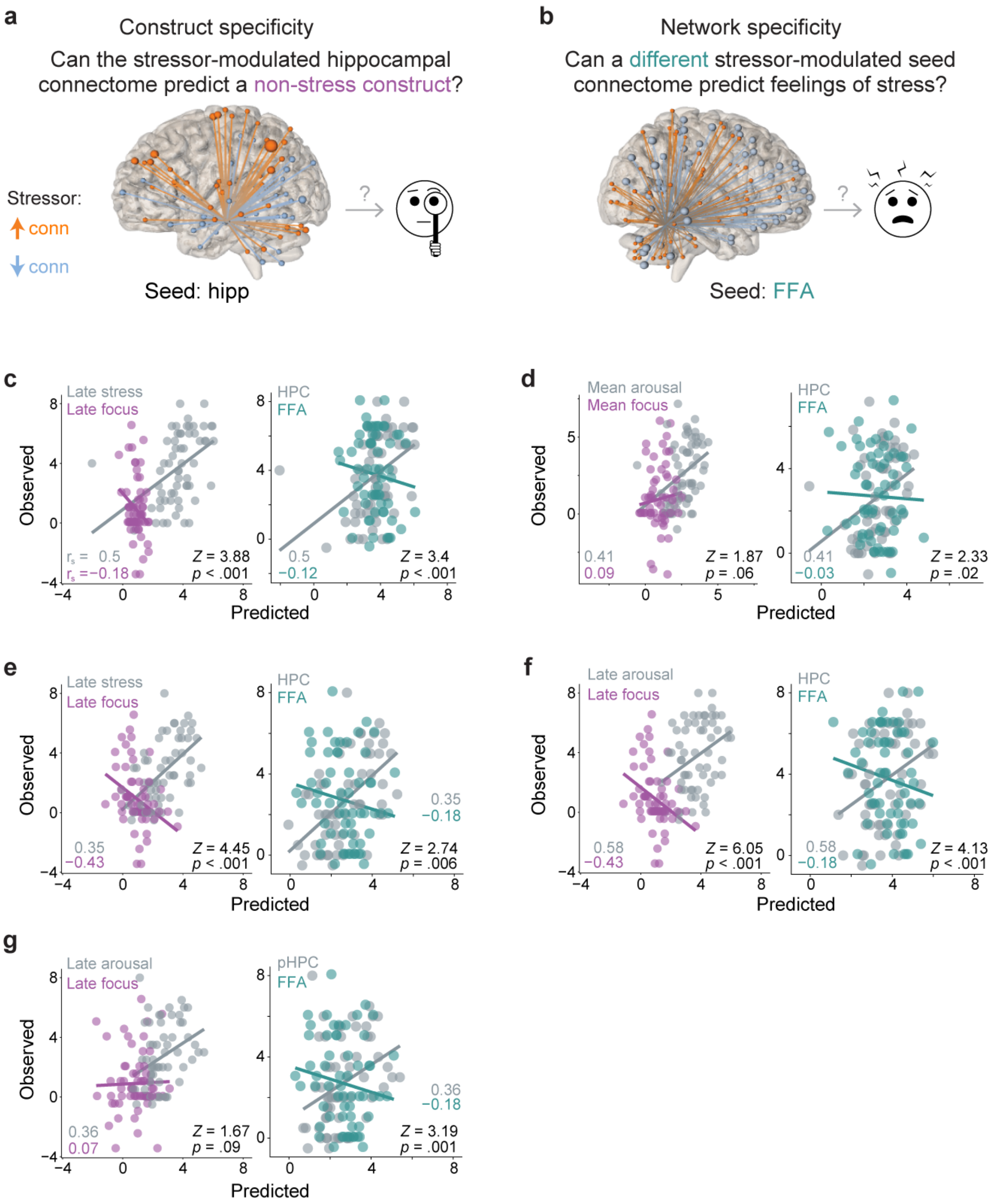

Supplementary Fig. 2. Construct and network specificity of all hippocampal models predicting the feeling of stress. **a-b,** Schematic of analyses. **c-g,** All predictive hippocampal and posterior hippocampal networks (also shown in Fig. 2b-i) demonstrate both construct and network specificity. Hippocampal network results from Fig. 2 are re-plotted in gray for reference. Left, construct specificity results showing that hippocampal networks can successfully predict stress-related feelings (shown in gray) but not ratings of focus (purple; analysis described in **a**). r_s = Spearman's correlation. Correlations near or below zero indicate model failure. Right, network specificity results, showing the ability of a control stressor-modulated network (fusiform face area seed) to predict the feeling of stress (analysis described in **b**). Steiger's *Z* (two-tailed) shows comparison of prediction success for hippocampal predictions of focus vs. stress (left) and hippocampal vs. FFA predictions of stress (right). Source data are provided as a Source Data file.

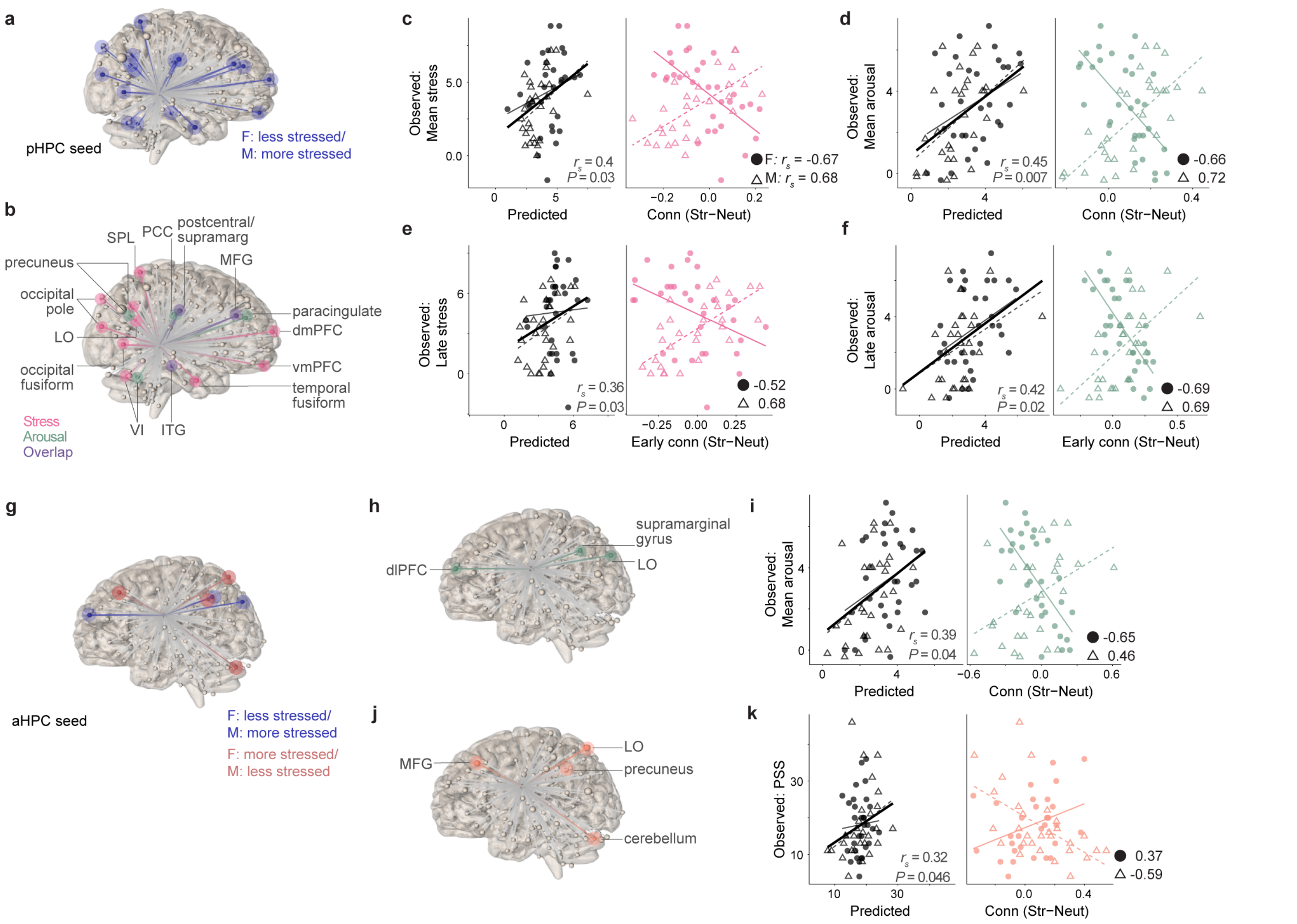

Supplementary Fig 3. Stressor-induced hippocampal connectivity networks predicting sex-specific feelings of stress. For each set of N-1 participants, Fisher-transformed hippocampal connectivity with each cluster was correlated with feelings of stress separately for male (M) and female (F) participants. For each cluster, M and F correlations between hippocampal connectivity and behavior were z-transformed and compared using two-tailed Fisher's z tests. Clusters where correlations with feelings of stress significantly differed between M and F participants were separated into F-/M+ and F+/M- networks, averaged, and used to build interaction models where behavior varied as a function of connectivity and sex. These models predicted feelings of stress in the left-out participant based on that individual's sex and hippocampal connectivity, with statistical significance assessed nonparametrically. Throughout figure, data from male participants is plotted as open triangles and female participants as closed circles. Source data are provided as a Source Data file.

a, Summary of networks identified from posterior hippocampal (pHPC) seed. **b-f,** pHPC networks predicting feeling less stressed (females) and more stressed (males), separately for ratings of stress (pink), arousal (green), or both (purple). **b,** Anatomical distribution of identified networks. **c-f,** Summary of predictive networks. Left, predictive power; Spearman's correlation (r_s) of model-predicted with observed ratings. *P* values determined nonparametrically (1000 iterations of randomly-shuffled feelings of stress). Right, correlation between overlap network strength and observed ratings, separately for female (filled circles) and male (open triangles) participants.

g, Summary of networks identified from anterior hippocampal (aHPC) seed. **h-i,** aHPC network predicting lower arousal in females and higher arousal in males. **j-k,** aHPC network predicting higher chronic stress (perceived stress scale, PSS) in females and lower chronic stress in males.

dmPFC = dorsomedial prefrontal cortex; ITG = inferior temporal gyrus; LO = lateral occipital cortex; MFG = medial frontal gyrus; PCC = posterior cingulate cortex; SPL = superior parietal lobule,

Supplementary Table 1. Participant demographics

	Number (%) participants
**Demographics**	
Gender (Female)	31 (51.7%)
Age	M = 29.6, SD = 8.61
**Psychiatric History**	
Any depression, anxiety, PTSD (current or lifetime)	16 (26.7%)
Depression	
Past month	1 (1.7%)
Lifetime	10 (16.7%)
Anxiety Disorder	
Past 6 months	5 (8.3%)
Lifetime	7 (10.6%)
PTSD	
Past 6 months	5 (8.3%)
Lifetime	6 (10%)

Anxiety disorders include: generalized anxiety disorder, social anxiety disorder, panic disorder, and medication-induced anxiety disorder. Psychiatric history was assessed using DSM-V criteria as determined by the Structured Clinical Interview for DSM-V (SCID-I)[1, 2] interviews conducted by trained Masters or doctoral-level research staff. Diagnoses made following SCID-5 criteria.

Supplementary Table 2. Overlapping stressor-modulated networks across hippocampal seeds

		Seed: Hipp				Seed: aHPC				Seed: pHPC			
		**X**	**Y**	**Z**	**Size**	**X**	**Y**	**Z**	**Size**	**X**	**Y**	**Z**	**Size**
**Frontal**													
Frontal pole	L	-23	52	30	40	-22	54	24	16	-23	50	30	18
	R	18	66	6	34					20	66	10	12
dlPFC	R	34	50	32	18					36	39	36	74
Frontal pole/vlPFC	R	17	48	-17	15					23	45	-16	37
MFG	L	-29	40	29	32					-33	37	30	67
Paracingulate/superior frontal gyrus	L	-2	39	37	57	-5	39	36	27				
OFC	L	-13	26	-20	24	-12	26	-21	23				
IFG	R	50	25	2	15	50	23	3	29				
Precentral/superior frontal gyrus	L	-22	-11	68	43	-23	-14	69	19	-23	-12	68	19
***Pre/postcentral gyrus**	**R**	**2**	**-36**	**75**	**19**	**2**	**-37**	**74**	**29**				
**Parietal**													
Postcentral gyrus	R	42	-24	33	30	46	-28	31	21				
Precentral gyrus	L	-8	-30	53	16					-8	-27	53	18
Posterior cingulate	L					-4	-30	28	17	-3	-26	30	18
Supramarginal/angular gyrus	R	48	-45	55	15	47	-43	55	51				
Supramarginal gyrus	L	-56	-50	41	106					-57	-46	44	60
Precuneus	L	-5	-52	50	25					-6	-52	53	17
Angular gyrus	R	51	-53	14	17	54	-55	15	75				
LO, superior	L	-57.5	-58.1	31	19	-57	-59	31	13				
	L	-34	-63	46	24	-36	-64	46	14	-29	-62	42	21
	L	-32	-68	57	16	-30	-68	57	39				
	R	35	-79	37	44	34	-78	34	25	34	-83	38	13
**Temporal**													
Temporal pole	R	50	9	-28	30	52	8	-26	32				
Planum polare/Heschl's gyrus	L	-46	-19	-1	16	-46	-18	0	30				
Superior temporal gyrus	R	45	-27	-3	19					41	-25	-6	12
PHC	R	16	-33	-17	40	13	-31	-20	14	22	-35	-15	21
MTG	R	64	-33	-14	16	61	-36	-16	21				
ITG	R	54	-39	-24	41	54	-39	-25	26				
Temporal occipital fusiform cortex	R	44	-47	-13	25					44	-50	-13	26
PHC, temporal fusiform	R					36	-2	-31	16	37	-6	-31	25
**Occipital**													
Lingual gyrus	L	-17	-52	-5	16	-17	-51	-5	17				
Intracalcarine cortex/lingual gyrus	R	25	-72	5	22					27	-68	1	13
LO, inferior	L	-50	-75	0	50					-49	-78	-1	20
LO, superior	L	-31	-87	30	26	-30	-81	34	18				
**Subcortical**													
Caudate	R	18	23	13	38	17	25	15	23	21	25	12	15
***PHC**	**R**	**22**	**3**	**-41**	**18**	**22**	**4**	**-41**	**17**				
Amygdala/temporal pole	L	-32	1	-19	26					-32	5	-19	33
***Hypothalamus**	**L**	**-8**	**0**	**-14**	**26**	**-11**	**1**	**-13**	**23**				
Putamen	L	-27	-10	11	15	-27	-10	10	18				
Putamen/thalamus	R	21	-13	16	17	26	-14	14	14				
Thalamus	L	-8	-15	6	22	-8	-15	6	19				
Hippocampus	R	20	-19	-18	82	21	-20	-19	25	22	-21	-19	34
Hippocampus/PHC	R	20	-19	-18	82	20	-22	-10	17				
**Cerebellum**													
VIIb	L	-40	-45	-49	22	-40	-45	-49	18				
***VIIb**	**R**	**29**	**-66**	**-47**	**23**	**29**	**-66**	**-47**	**20**				
Vermis VI	R	2	-70	-24	19	1	-69	-25	28				
Crus I	R	17	-82	-30	23	17	-82	-30	14				
Crus I	R	8	-83	-23	25	0	-83	-25	50				
Crus I/II	L	-40	-46	-39	19					-42	-48	-43	64
Crus II	L	-5	-85	-26	39	0	-83	-25	50	-9	-86	-27	48

Significant stressor-modulated connectivity networks (voxelwise $p < .001$, cluster $\alpha = .05$) using full, anterior (aHPC) and posterior (pHPC) hippocampus as seeds. Clusters shown have $\geq$1 voxel overlap across seed maps. Clusters identified as belonging to predictive networks across multiple seeds are highlighted in bold (see Fig. 5).

Supplementary Table 3. Comparison of leave-one-out to *k*-fold cross-validation results.

	Predictive Power					Identified Networks		
	LOO		10-fold			LOO	10-fold	
Network	r	p	r	Cohen's d	p (est)	Network clusters	% folds (m)	Ranking
Hippocampus								
Late stress (+)	.498	.007	.29	.90	D = .77, *p* < .001	R PHC	100%	1
						L hypothalamus	84%	2
						R MTG	76.8%	3
Late stress (-)	.35	.04	.13	.49	D = .53, *p* < .001	L postcentral/ precuneus	81.77%	1
						R vermis	79.74%	2
Mean arousal (+)	.41	.001	.25	.78	D = .69, *p* < .001	R ITG	92.5%	1
						L hypothalamus	90.5%	2
Late arousal (-)	.58	.002	.45	1.41	D = .96, *p* < .001	L dlPFC	100%	1
						R vermis	99.98%	2
						L post putamen	73.6%	3
Posterior Hippocampus								
Late arousal (-)	.36	.045	.26	.83	D = .75, *p* < .001	L precentral	97.9%	1
						L MFG	97.4%	2
						R crus I	91.8%	3

To account for potential bias due to the leave-one-out (LOO) cross-validation procedure, we implemented a 10-fold cross-validation approach. LOO results (Spearman's rho, non-parametric p values, and identified clusters) are provided for reference.

k-fold: Predictive power. In each fold, the left-out subset (N = 6) was constrained such that <70% of left-out participants were of any one sex (as predictive networks can vary by sex; see main text). The model was trained (on N = 54 participants) and tested on the left-out subset as described (Methods, Fig 2a). This process was repeated 1000x with participants randomly assigned into folds to avoid bias in fold assignment. Predictive power was taken as the average Spearman's rho value across all iterations, and effect size was computed by comparing the distribution of Spearman's rho from models predicting true stress ratings to a null distribution (1000 iterations of models predicting randomly shuffled stress ratings) as in [3]. All comparisons resulted in medium or large effect sizes. P value was approximated using Kolmogorov-Smirnov tests, although the emphasis for robustness and interpretation should be on the effect size[3].

k-fold: Network identification. Identified networks were quantified in two ways. We first computed the frequency (% of folds) with which each cluster was selected using 10-fold cross-validation across the 1000 iterations. We then used this frequency to rank-order all clusters. As shown in Table 1, the vast majority of clusters identified using LOO were also the most frequently selected using 10-fold cross-validation.

Supplementary Table 4. Clusters identified as part of predictive hippocampal networks.

Network		Cluster	X	Y	Z	Size (voxels)	Str v N *Z* [SEM]
Hippocampus							
Positive	L	hypothalamus	-8	0	-14	26	-3.96 [.11]
	R	parahippocampal cortex	22	3	-41	18	-3.83 [.07]
	R	inferior temporal gyrus	54	-39	-24	41	-3.95 [.08]
	R	middle temporal gyrus	64	-33	-14	16	-4.04 [.12]
Negative	L	dlPFC	-29	49	29	32	4.01 [.09]
	L	postcentral gyrus/ precuneus	-7	-44	64	14	3.85 [.12]
	L	post putamen	-29	-20	13	14	3.81 [.14]
	R	vermis	1	-64	-32	15	4.21 [.12]
Posterior hippocampus							
Negative	L	middle frontal gyrus	-40	16	54	12	-3.94 [.2]
	L	precentral gyrus	-7	-15	71	16	4.2 [.14]
	R	cerebellar crus I	43	-44	-34	22	4.03 [.11]

All clusters were defined as showing significantly different background connectivity with the hippocampal seed region during Stress relative to Neutral conditions (voxelwise $p < .001$; $\alpha = .05$). MNI coordinates shown for center of mass of identified clusters.

Supplementary Table 5. Functional connectivity/behavior relationships for each full network and strongest individual cluster

Seed region	Model	Best cluster	Seed connectivity with **network** x stress ratings	Seed connectivity with **cluster** x stress ratings	Steiger's *Z* (abs)
sCPM networks					
Hippocampus	Mean connectivity/ Mean arousal [+]	R ITG	0.46	0.33	1.38†
	Early connectivity/ Late stress [+]	R PHC	0.53	0.40	1.25
	Early connectivity/ Late stress [-]	L postcentral gyrus/precuneus	-0.45	-0.3	1.83*
	Early connectivity/ Late arousal [-]	L post putamen	-0.65	-0.29	3.02**
Posterior hippocampus	Early connectivity/ Late arousal	R crus I	-0.49	-0.32	1.67*

Functional connectivity/behavior relationships for each full network and strongest individual cluster. Spearman's correlations used in *Z* test to compare dependent correlations (Eq 3 & 10)[4]. †$p < .1$, *$p < .05$, **$p < .01$, one-tailed.

References

1. First MB, Williams JBW, Karg RS, Spitzer RL. *Structured Clinical Interview for DSM-5, Research Version*. American Psychiatric Association (2015).

2. American Psychiatric Association. *Diagnostic and statistical manual of mental disorders*, 5th ed edn. Arlington, VA (2013).

3. Rudolph MD*, et al.* Maternal IL-6 during pregnancy can be estimated from newborn brain connectivity and predicts future working memory in offspring. *Nat Neurosci* **21**, 765-772 (2018).

4. Steiger JH. Tests for comparing elements of a correlation matrix. *Psychol Bull* **87**, 245-251 (1980).
