# Supplementary material for: Hippocampal seed connectome-based modeling predicts the feeling of stress
Source: Nat Commun. 2020 May 27;11:2650. doi: 10.1038/s41467-020-16492-2 (PMC7253445; doi:10.1038/s41467-020-16492-2)
Supplement: Supplementary file 3 — Reporting Summary [file 41467_2020_16492_MOESM3_ESM.pdf]

nature research

Corresponding author(s): Rajita Sinha

Last updated by author(s): May 4, 2020

Reporting Summary

Nature Research wishes to improve the reproducibility of the work that we publish. This form provides structure for consistency and transparency in reporting. For further information on Nature Research policies, see our Editorial Policies and the Editorial Policy Checklist.

Statistics

For all statistical analyses, confirm that the following items are present in the figure legend, table legend, main text, or Methods section.

n/a	Confirmed	
☐	☒	The exact sample size (*n*) for each experimental group/condition, given as a discrete number and unit of measurement
☐	☒	A statement on whether measurements were taken from distinct samples or whether the same sample was measured repeatedly
☐	☒	The statistical test(s) used AND whether they are one- or two-sided<br>*Only common tests should be described solely by name; describe more complex techniques in the Methods section.*
☐	☒	A description of all covariates tested
☐	☒	A description of any assumptions or corrections, such as tests of normality and adjustment for multiple comparisons
☐	☒	A full description of the statistical parameters including central tendency (e.g. means) or other basic estimates (e.g. regression coefficient) AND variation (e.g. standard deviation) or associated estimates of uncertainty (e.g. confidence intervals)
☐	☒	For null hypothesis testing, the test statistic (e.g. *F*, *t*, *r*) with confidence intervals, effect sizes, degrees of freedom and *P* value noted<br>*Give P values as exact values whenever suitable.*
☒	☐	For Bayesian analysis, information on the choice of priors and Markov chain Monte Carlo settings
☒	☐	For hierarchical and complex designs, identification of the appropriate level for tests and full reporting of outcomes
☐	☒	Estimates of effect sizes (e.g. Cohen's *d*, Pearson's *r*), indicating how they were calculated

Our web collection on statistics for biologists contains articles on many of the points above.

Software and code

Policy information about availability of computer code

Data collection	Stimuli were presented using EPrime software (2.0)
Data analysis	fMRI data were pre-processed using FSL 6.0.1 and AFNI 18.3.08. Group-level maps were computed using AFNI's 3dLME. Analyses of behavior and predictive models were generated using R 3.4.1, with predictive models based on publicly available CPM code written for MATLAB (https://github.com/YaleMRRC/CPM).

For manuscripts utilizing custom algorithms or software that are central to the research but not yet described in published literature, software must be made available to editors and reviewers. We strongly encourage code deposition in a community repository (e.g. GitHub). See the Nature Research guidelines for submitting code & software for further information.

Data

Policy information about availability of data

All manuscripts must include a data availability statement. This statement should provide the following information, where applicable:
- Accession codes, unique identifiers, or web links for publicly available datasets
- A list of figures that have associated raw data
- A description of any restrictions on data availability

The data that support the findings of this study are available from the corresponding author upon reasonable request.

Field-specific reporting

Please select the one below that is the best fit for your research. If you are not sure, read the appropriate sections before making your selection.

☐ Life sciences ☒ Behavioural & social sciences ☐ Ecological, evolutionary & environmental sciences

For a reference copy of the document with all sections, see nature.com/documents/nr-reporting-summary-flat.pdf

Behavioural & social sciences study design

All studies must disclose on these points even when the disclosure is negative.

Study description	This is a quantitative experimental study that investigated whether functional hippocampal networks could significantly predict the subjective feeling of stress.
Research sample	Participants were a representative sample of individuals from the New Haven community. 60 participants (31 female) participated in the experiment (mean age = 28.73, range = 19-50 years old). As the goal of this study was to predict subjective stress from brain responses, participants were excluded who reported factors that could significantly modulate the physiological or emotional stress response. These exclusion criteria included: 1) current criteria for any moderate/severe substance use disorder; 2) current opiate use; 3) psychosis/severe psychiatric disability; 4) significant medical conditions; 5) regular use of medications that could interfere with the stress response; and 6) for female participants, pregnant or nursing. For MRI safety, participants were also excluded who reported 6) claustrophobia or ferromagnetic metal in the body (MRI safety).
Sampling strategy	A convenience sampling procedure was used. Sample size was determined based on prior work showing that significant stressor-modulated brain responses could be observed using this protocol for N = 30 participants (Sinha et al 2016 PNAS). To account for sex differences in stress responses (Goldfarb, Seo & Sinha 2019 Neurobio Stress), we doubled this sample size to have equivalent power for male (N = 29) and female participants (N = 31).
Data collection	Stimuli were presented using E-Prime software (2.0) and projected onto a screen that participants viewed during the MRI scan using a mirror attached to the head coil. A research assistant and MR technician were present during data collection. These individuals were aware of the experimental conditions but not the study hypothesis.
Timing	Nov 2015 - Jul 2018
Data exclusions	One run was excluded due to excessive motion (>1.5mm mean absolute frame-to-frame displacement) based on pre-established criteria.
Non-participation	No participants dropped out.
Randomization	Participants were not allocated into experimental groups

Reporting for specific materials, systems and methods

We require information from authors about some types of materials, experimental systems and methods used in many studies. Here, indicate whether each material, system or method listed is relevant to your study. If you are not sure if a list item applies to your research, read the appropriate section before selecting a response.

Materials & experimental systems

n/a	Involved in the study	
☒	☐	Antibodies
☒	☐	Eukaryotic cell lines
☒	☐	Palaeontology and archaeology
☒	☐	Animals and other organisms
☐	☒	Human research participants
☒	☐	Clinical data
☒	☐	Dual use research of concern

Methods

n/a	Involved in the study	
☒	☐	ChIP-seq
☒	☐	Flow cytometry
☐	☒	MRI-based neuroimaging

Human research participants

Policy information about studies involving human research participants

Population characteristics	See above
Recruitment	Participants were recruited from the community using flyers and web-based advertisements (including social media). Given the range of subjective stress responses reported, we do not believe that selection biases impacted the fidelity of the results presented in the manuscript.
Ethics oversight	The Yale School of Medicine Institutional Review Board approved all procedures.

Note that full information on the approval of the study protocol must also be provided in the manuscript.

Magnetic resonance imaging

Experimental design

Design type	Task-state fMRI, block design
Design specifications	Participants passively viewed 132 unique images in Stressor and Neutral conditions using a block design. There was a recovery period between conditions (~5 min) during which participants were provided progressive relaxation instructions. Each condition contained 8 contiguous runs (66s each): 2 baseline (5s gray screen, 1s inter-stimulus interval [ISI]) followed by 6 image runs (5s image, 1s ISI). During each ISI, a black screen was presented with a white central fixation point.
Behavioral performance measures	After every run, participants rated their levels of stress (1: not at all stressed while viewing the pictures, 9: extremely stressed), arousal (1: calm/relaxed, 9: highly aroused or excited), and focus (1: not at all, 9: very well) using an MRI-compatible button box (responses self-paced). Prior to the scan, participants completed practice trials outside the fMRI scanner, viewing 4 unique images (not repeated during the experiment) and learned to use the button box to make ratings.

Acquisition

Imaging type(s)	Functional and structural
Field strength	3T
Sequence & imaging parameters	Structural data were acquired using a sagittal high-resolution T1-weighted 3D MPRAGE sequence (2400ms TR, 1.96ms TE, flip angle: 8°, FOV: 256x256, 208 slices, 1mm3 isotropic voxels). Functional data were acquired using a multiband (5 slices/RF pulse) gradient EPI sequence (1000ms TR, 30ms TE, flip angle: 55°, FOV: 220×220, 75 slices, interleaved acquisition, 2mm3 isotropic voxels; 4s dummy run pre-acquisition).
Area of acquisition	Whole brain

Diffusion MRI ☐ Used ☒ Not used

Preprocessing

Preprocessing software	Scans were preprocessed using FSL 6.0.1 and AFNI 18.3.08. Data were high-pass filtered at 0.01 Hz to remove low-frequency drifts in signal, and runs with excessive head motion (defined a priori as >1.5mm absolute mean frame-to-frame displacement, MCFLIRT), were excluded (1 run from 1 participant).
Normalization	Model residuals (see "Noise and artifact removal" below) were aligned to a reference functional scan and then to the participant's high-resolution anatomical scan using FSL's boundary based registration (Greve & Fischl 2009). Images were then warped to MNI space and visually inspected for alignment. Afterward, they were smoothed to 6mm FWHM (using AFNI's 3dBlurToFWHM).
Normalization template	Data were normalized to MNI-152 space
Noise and artifact removal	A general linear model (GLM) was conducted per run to control for motion and covariates of no interest (FEAT; Woolrich et al 2001). Regressors included: 6 linear estimated motion parameters, white matter, cerebrospinal fluid, and global mean signal timeseries (each plus temporal derivatives) and stick function regressors for nonlinear motion outliers. To focus on background connectivity, we removed trial-evoked signal (image on/offset modeled using a boxcar convolved with a double-gamma HRF, plus temporal derivatives).
Volume censoring	N/A

Statistical modeling & inference

Model type and settings	The average time course of responses within each hippocampal ROI (defined anatomically per participant) was computed from the preprocessed data, separately per baseline/early/mid/late epochs (132 sec each). ROI timeseries were each

correlated with the timeseries of all voxels throughout the brain and resulting r maps were Fisher z-transformed. To assess changes induced by stress (and enable us to interpret relative correlation directionality), differences in connectivity during each image epoch (early/mid/late; 528 sec total) relative the immediately preceding baseline were computed.
These differential functional connectivity maps were then entered into a second-level linear mixed effects model (3dLME) with Condition, Epoch and Sex as fixed effects and participant as a random effect.

Effect(s) tested

The contrast used to define significant stressor-modulated clusters, which would be used in the seed connectome-based predictive models, was the Condition contrast of Stressor vs Neutral.

Specify type of analysis: ☒ Whole brain ☐ ROI-based ☐ Both

Statistic type for inference
(See Eklund et al. 2016)

For voxelwise p<.001, we used bi-sided first-nearest neighbor clustering to determine the cluster threshold for α = .05. This cluster correction was conducted using the latest 3dClustSim (Cox et al 2017).

Correction

See above

Models & analysis

n/a	Involved in the study
☐	☒ Functional and/or effective connectivity
☒	☐ Graph analysis
☒	☐ Multivariate modeling or predictive analysis

Functional and/or effective connectivity

The average time course of responses within each hippocampal ROI was computed from the preprocessed data (separately per baseline/early/mid/late epochs; 132 sec each). ROI timeseries were each correlated with the timeseries of all voxels throughout the brain (Pearson's correlation) and resulting r maps were Fisher z-transformed.
